# Retraction: Immobilization of metribuzin-degrading bacteria on biochar: Enhanced soil remediation and bacterial community restoration

**DOI:** 10.3389/fmicb.2024.1441699

**Published:** 2024-06-11

**Authors:** Frontiers Editorial Office

The journal retracts the 2023 article cited above.

Following publication, concerns were raised regarding the contributions of the authors of the article. Our investigation, conducted in accordance with Frontiers policies, confirmed a serious breach of our authorship policies and of publication ethics; the article is therefore retracted.

This retraction was approved by the Chief Executive Editor of Frontiers. The authors received a communication regarding the retraction and had a chance to respond. This communication has been recorded by the publisher.

